# Exopolysaccharides of Lactic Acid Bacteria: Production, Purification and Health Benefits towards Functional Food

**DOI:** 10.3390/nu14142938

**Published:** 2022-07-18

**Authors:** Helena Mylise Sørensen, Keith D. Rochfort, Susan Maye, George MacLeod, Dermot Brabazon, Christine Loscher, Brian Freeland

**Affiliations:** 1 School of Biotechnology, Dublin City University, D09 DX63 Dublin, Ireland; christine.loscher@dcu.ie (C.L.); brian.freeland@dcu.ie (B.F.); 2 I-Form, Advanced Manufacturing Research Centre, Dublin City University, D09 DX63 Dublin, Ireland; dermot.brabazon@dcu.ie; 3 School of Nursing, Psychotherapy and Community Health, Dublin City University, D09 DX63 Dublin, Ireland; keith.rochfort@dcu.ie; 4 Dairygold Co-Operative Society Limited, Clonmel Road, Co. Cork, P67 DD36 Mitchelstown, Ireland; smaye@dairygold.ie (S.M.); gmacleod@dairygold.ie (G.M.)

**Keywords:** exopolysaccharides, lactic acid bacteria, functional food, health benefits, postbiotics

## Abstract

Lactic acid bacteria (LAB) are capable of synthesising metabolites known as exopolysaccharides (EPS) during fermentation. Traditionally, EPS plays an important role in fermented dairy products through their gelling and thickening properties, but they can also be beneficial to human health. This bioactivity has gained attention in applications for functional foods, which leads them to have prebiotic, immunomodulatory, antioxidant, anti-tumour, cholesterol-lowering and anti-obesity activity. Understanding the parameters and conditions is crucial to optimising the EPS yields from LAB for applications in the food industry. This review provides an overview of the functional food market together with the biosynthesis of EPS. Factors influencing the production of EPS as well as methods for isolation, characterisation and quantification are reviewed. Finally, the health benefits associated with EPS are discussed.

## 1. Introduction

The foods that we consume have ascended from being appetizing and nutritious as their primary requirements to now being an active tool used to improve human health through added functionality [1]. Diet is an important factor in general human health and is considered one of the first lines of defence to prevent many diseases including cancer [2], heart disease [3] and osteoporosis [4]. With an increasing interest in the connection between food and health, products characterized as functional foods are growing in popularity.

Functional foods have no universally agreed definition; however, they are often defined as foods that provide a variety of health benefits when consumed. Japan was the first country to recognise functional food as a unique category and defined it as Food for Specified Health Use (FOSHU) [5]. In Europe, the term has been described by The European Commission’s Concerted Action on Functional Food Science in Europe (FuFoSE), coordinated by International Life Science Institute (ILSI):

*“A food can be regarded as ‘functional’ if it is satisfactorily demonstrated to affect beneficially one or more target functions in the body, beyond adequate nutritional effects, in a way that is relevant to either an improved state of health and well-being and/or reduction of risk of disease. Functional foods must remain foods and they must demonstrate their effects in amounts that can normally be expected to be consumed in the diet: they are not pills or capsules, but part of a normal food pattern”* [6].

Functional food can be classified into four categories based on this definition: conventional foods, modified foods, foods for special dietary requirements and medicinal foods [7]. Whole foods including grains, fish, fruits, and vegetables are examples of conventional foods that naturally include bioactive food components, which also act as a foundation for the next three functional food groups. Foods that have been enhanced, supplemented, or fortified are known as modified foods. Milk enriched with useful components such as minerals and vitamins [8] and bread enriched with folic acid [9] are examples of this category. Foods for special dietary requirements include commercially available foods such as infant formula [10], gluten-free products and lactose-free dairy products to accommodate food intolerances and allergies [7]. The last group of medical foods differ from foods for special dietary requirements by being strictly administered under the consultation and supervision of a physician. These foods are specially formulated to be supplemented/free of one/several compound/s for those of a particular medical disposition (e.g., phenylalanine-free foods for patients with phenylketonuria) [11].

As such, there is growing interest in the characterisation and subsequent implementation of bioactive ingredients into foods in order to provide populations with sustenance which meets medically defined criteria. One novel approach gaining attention is fermentates; a powdered preparation derived from fermentation reactions that can consist of the fermenting microorganisms themselves or metabolites and bioactive components excreted in the fermentation broth.

### 1.1. Market Value of Functional Foods

The functional foods market is experiencing large growth in Europe. The market itself is experiencing growing attention with an increase of publications related to functional food every year as well as continuous development of new products released to the market each year [12]. Several health-oriented factors have contributed to this market growth, including an increase in life expectancy, an increase in the cost of health care, and a general focus on nutrition for quality of life and well-being [13].

**Figure 1:**
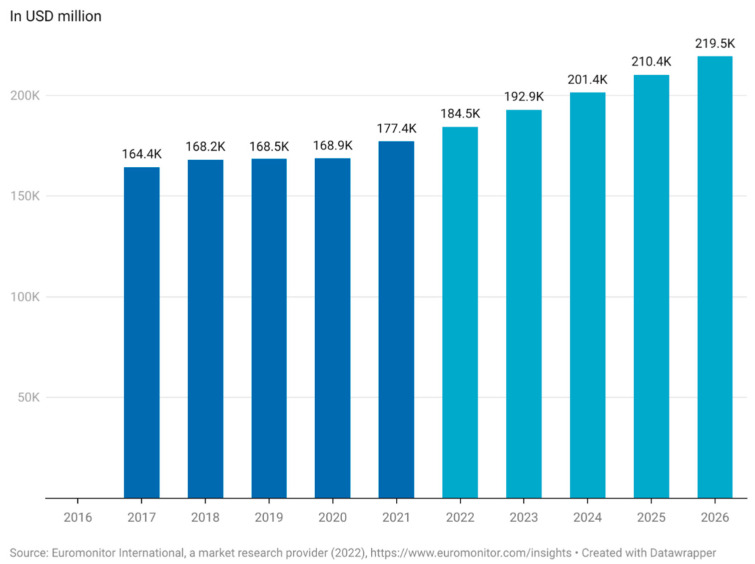
Market size of functional food worldwide (Euromonitor International, a market research provider (2022), (in USD million). This figure was made by the authors with raw data obtained from Euromonitor. Approval for use of data has been obtained from Euromonitor.

The lack of definition of functional foods complicates the estimation of the actual market value for functional foods [14]. Euromonitor has estimated the worth of the global functional food market to be USD 177.4 billion in 2021 and expects the market to reach USD 219.5 billion by 2026 (Figure 1). This equates to an annual increase in the market of 4.3–4.5%. The functional food market saw a steep increase between 2020 and 2021 of 5% compared to just 0.3% from 2019 to 2020 (Figure 1) [15]. This is most likely due to the increased focus on health caused by the COVID-19 pandemic but is a trend that appears to continue through the rest of the decade. According to a report made by Euromonitor in 2021, functional food is now one of the top emerging trends among consumers [16].

The fortification of foodstuffs with vitamin D is an example of functional foods that gained attention during the pandemic, as vitamin D insufficiency has been related to an increased risk of respiratory tract infections [17].

**Figure 2:**
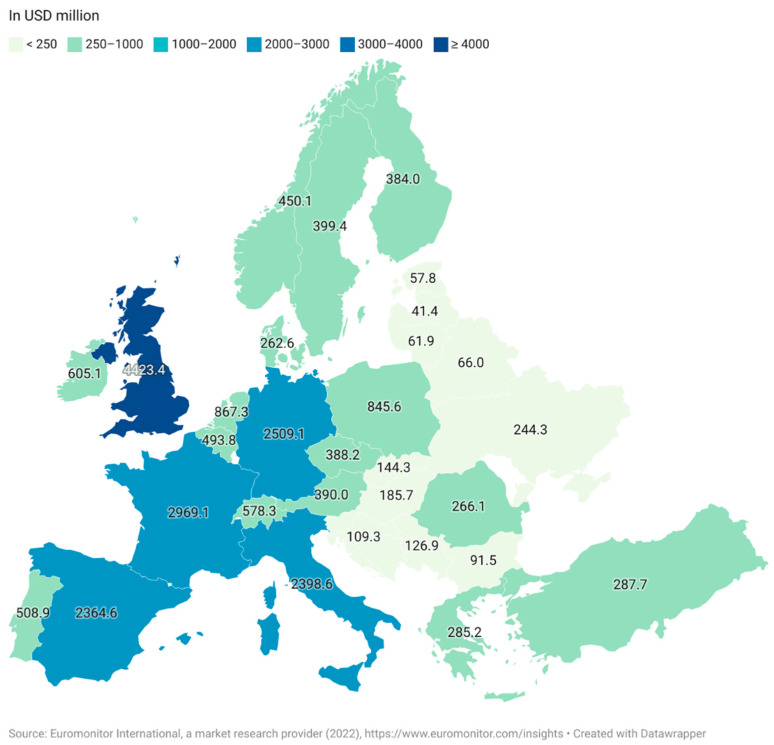
Retail sale value of functional foods in Europe in 2021 (Euromonitor International, a market research provider (2022), (in USD million).

The three most significant regions for functional food sales are the USA, Europe and Japan. As a whole, the European market is less developed than in Japan and the USA due to the historically legislative and regulative frameworks associated with the approval of products in the EU and also a regional distrust of marketed health benefits of processed foods within the region [18]. The UK is the largest market in Europe as of 2021, followed by Germany and France (Figure 2). The market for functional foods is higher in Western Europe than in Eastern Europe, although Eastern Europe has established itself as an emerging market [19]. The estimated market value for the Eastern European market was USD 5213 million while the Western European market had an estimated value of USD 23,033 million. Both markets saw a steep increase between 2020 and 2021 with 6% for Eastern Europe and 7% for Western Europe [15].

### 1.2. Current Commercial and Research Interest

**Figure 3:**
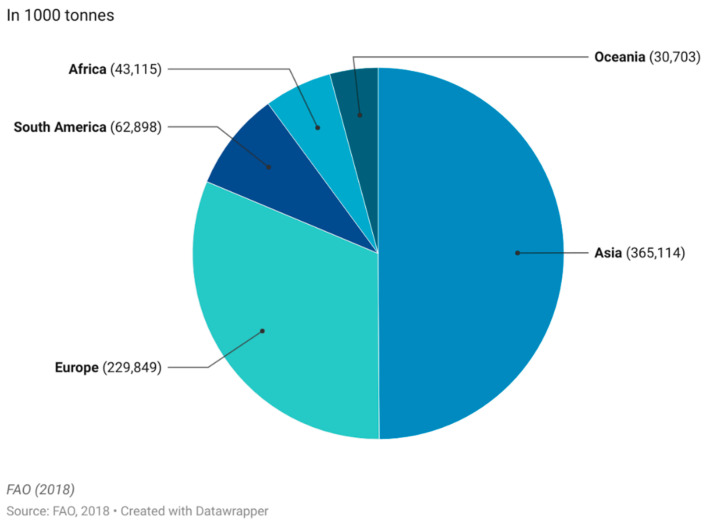
Worldwide production quantity of milk products (excl. butter) in 2018. This figure was made by the authors with raw data from FAO.

The commercial market of functional foods is complex as it varies with region, but one market of wide interest is the functional dairy market [14]. Dairy products make up a large portion of the market and are estimated to account for 33% of the entire global market [20]. The main reason for this market share is the natural presence of dairy products in a balanced daily diet, as they organically represent a rich source of macromolecular nutrients, vitamins and minerals with known benefits to human health. Adding extra nutritional content to milk-based products simply means modifying or enriching the already naturally healthy base [21]. Milk products (excluding butter) are the fourth largest food group produced worldwide with an annual worldwide production of 857 million tonnes produced, with Asia and Europe being the largest production continents (Figure 3).

The three biggest food categories are sugar cane (1.93 billion tonnes), maize and products (1.13 billion tonnes) and vegetable (other) (960 million tonnes).

One approach to enriching foods is through fermentates which are defined as a powdered preparation derived from fermentation that can consist of either the whole lysed microorganisms themselves, metabolites, or bioactive components also known as postbiotics [22]. Promising postbiotics known as EPS will be explored in this review, including methods for production, isolation purification, and quantification. Several health benefits have been associated with EPS including increased prebiotic activity, improved digestion, immunostimulatory, antiviral, antioxidant, anti-tumoral and cholesterol-lowering properties [23,24,25,26,27,28]. This alludes to the promising potential of LAB-produced EPS as a functional food ingredient. The EPS produced by LAB varies greatly in structure, and understanding these differences is key to assessing the functionality of EPS.

### 1.3. Exopolysaccharides as a Functional Food Ingredient

The microbial cell envelope is composed of glycan molecules that are either capsular and linked tightly to the cell surface, or in the form of EPS that can either be loosely attached or excreted into the environment of the cells. The ability to produce and excrete EPS is exhibited by many species of bacteria including LAB, propionibacteria and bifidobacteria during growth [29]. EPS harbours a diverse role in the bacterial environment mostly related to the protection of the cell against environmental factors such as desiccation, pH, osmotic pressure, light, metal ions, bacteriocins, phagocytosis, protozoa and toxic compounds [30,31,32,33]. EPS can also function as a carbon source for some organisms, but most bacteria that produce EPS do not have genes encoding EPS degradation and are therefore not capable of catabolising the polysaccharides [34].

When utilized as a functional food ingredient, EPS acts as a postbiotic. Postbiotics are defined as metabolic products released into the fermentation matrix or at cell lysis that can confer health benefits and also includes vitamins, peptides, acids and proteins in addition to EPS [35]. Postbiotics are advantageous over probiotics as a food ingredient, as it not required for the cells to be viable and therefore circumvent some of the processing, storage and shelf-life challenges of probiotics [35,36]. Prebiotics is another type of food ingredient and is defined by Gibson and Roberfroid (1995) as “*a nondigestible food ingredient that beneficially affects the host by selectively stimulating the growth and/or activity of one or a limited number of bacteria in the colon, and thus improves host health*” [37]. These non-digestible substrates include oligosaccharides, dietary fibres, polyphenols and short-chain fatty acids [38]. In conclusion, prebiotics act as the substrate for the commensal gut microbiome, probiotics constitute the live cell fraction and postbiotics refer to the metabolites excreted by bacteria during fermentation.

When EPS is added to food products, the matrix of the product is affected, resulting in changes to the texture, rheology, and viscosity. The degree and in what way EPS will have an influence on the physiochemical properties of food products will depend on the molecular weight, charge, and structure of the polymer [39,40]. Therefore, when applied as an additive, EPS has the potential to increase creaminess, viscosity, and thickness, decrease fat content, and replace emulsion, thickening, and stabilizer agents [40]. A study found that with the increasing addition of LAB-derived EPS an increase in the zeta potential of sodium caseinate could be observed. This would indicate the potential for increased stability of dairy products containing sodium caseinate in a dose-dependent manner of EPS [41].

### 1.4. Exopolysaccharide Structures and Biosyntheses

The structure of EPS can be divided into two different groups: homopolysaccharides and heteropolysaccharides. The homopolysaccharide type is comprised entirely of a single type of monosaccharide, whereas heteropolysaccharides consist of multiple types of monosaccharides [34]. Owing to these differences, the size profile of each type can be very different with the molecular weight of homopolysaccharides lying within a range of 10 kDa and 6000 kDa, while heteropolysaccharides vary more in molecular weight between 10 kDa and 10,000 kDa [42,43].

**Figure 4:**
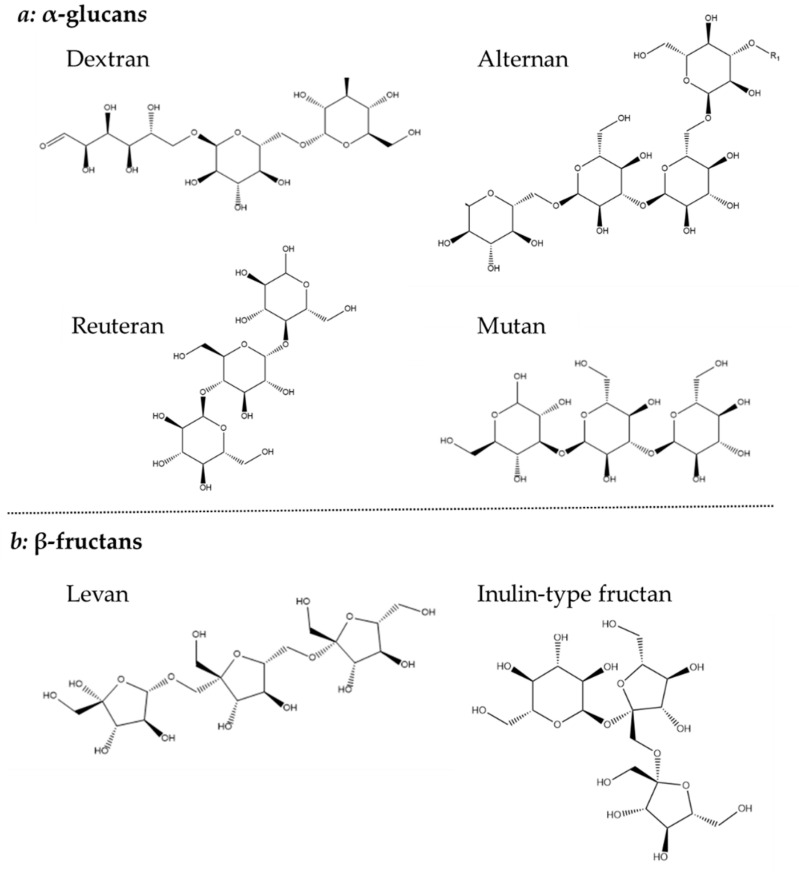
Chemical structure of (**a**) α-glucans and (**b**) β-fructans.

Homopolysaccharides are largely constructed from sucrose, by the polymerization of glucose or fructose, and have been further divided into four subgroups: α-glucans, β-glucan, β-fructans and α-galactan [44]. Both glucan types consist solely of glucose, but their different linkages make it possible to further distinguish their structure. The α-glucan type homopolysaccharide is linked with either α-1,2, α-1,3 α-1,4 or α-1,6 or glycosidic bonds and is classified as dextrans (α-1,6), alternans (1,3 α and α-1,6), mutans (1,3 α and α-1,6) and reuterans (1,4 and α-1,6). While four subtypes of α-glucans are known, only one β-glucan is described which is linked with β-1,2, β-1,3 and β-1,4 glycosidic bonds (Figure 4) [44,45].

The β-fructans contain units of fructose linked with either β-2,1 or β-2,6 and osidic bonds, which are divided into inulin-type (β-2,1) and levan (β-2,6). Finally, the less common β-galactan is comprised of galactose units linked with either β-1,3 or β-1,6 [44]. Both glucans, fructans and galactans are produced by LAB belonging to the genera of *Lactobacillus*, *Streptococcus* and *Leuconostoc* (Figure 4) [34].

**Figure 5:**
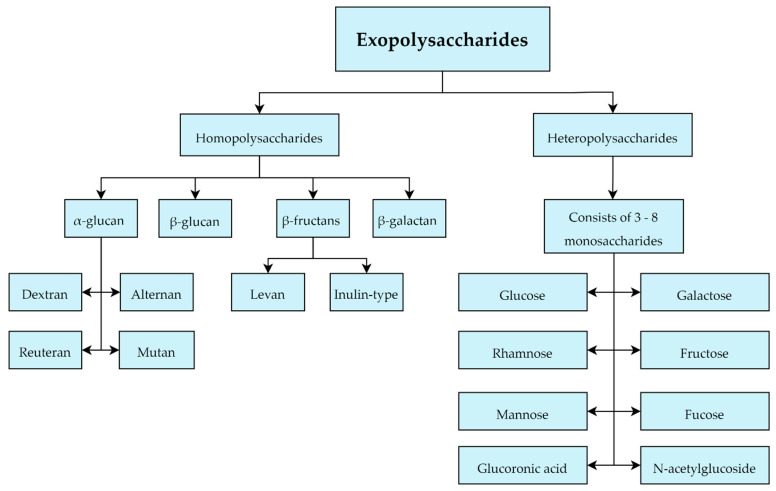
Classification of exopolysaccharides produced by *Lactobacillus* sp.

Most of the EPS produced by LAB belongs to the group of heteropolysaccharides which are typically composed of 3–8 units of glucose, galactose or rhamnose typically. However, they can also contain other monosaccharides including fructose, mannose, fucose, glucuronic acid and *N*-acetylglucosides, and additionally isoform-specific modifications to the monosaccharides that comprise them in the form of acetyl and phosphate groups (Figure 5) [45,46]. In this work, 201 LAB strains were evaluated in terms of heteropolysaccharides production with the following LAB strains evaluated for EPS production: *Lactobacillus. delbrueckii* subsp. *bulgaricus*, *L. delbrueckii* subsp *lactis*, *L. helveticus*, *L. casei*, *L. paracasei*, *L. rhamnosus*, *Enterococcus. faecalis*, *E. faecium* and *Streptococcus. thermophilus* [47]. This study emphasized the large structural diversity and molecular weight differences (8 kD to 5000 kD) of heteropolysaccharides [47].

**Figure 6:**
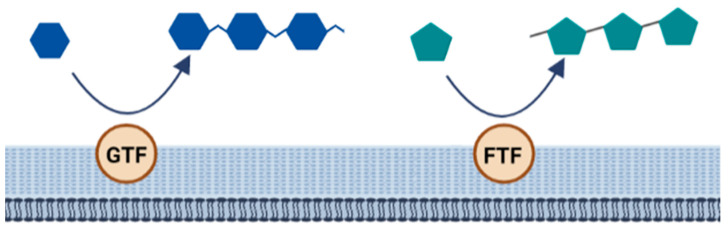
Biosynthesis of homopolysaccharides.

The biosynthesis of homopolysaccharides is carried out by extracellular enzymes called glycansucrases that are anchored to the cell wall. These are further divided into glycosyltransferase (GTF) and fructosyltransferase (FTF). GTF catalyzes the transfer of glucose while FTF catalyzes the transfer of fructose to a growing chain of homopolysaccharides. In addition, these enzymes are specific to forming different types of linkages [48] (Figure 6).

**Figure 7:**
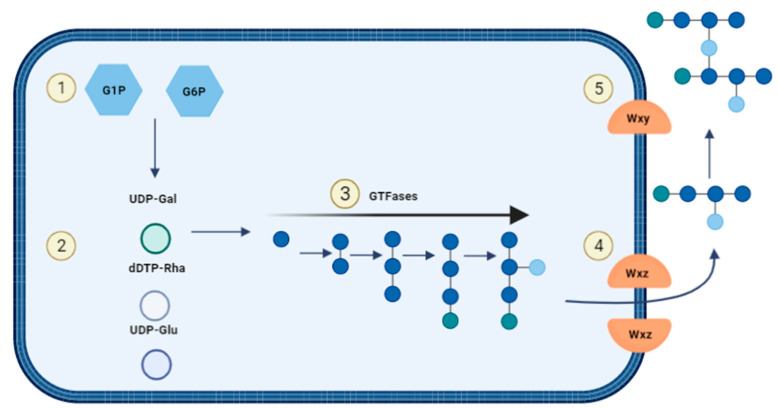
Biosynthesis of heterosaccharides.

For heteropolysaccharide biosynthesis in LAB, the Wzx/Wzy pathway is utilized which consists of five steps (Figure 7): (1) Saccharides will be transported into the cell and are phosphorylated to either glucose-1-phosphate or glucose-6-phosphate. (2) The sugar nucleotides uridine-diphosphate-glucose (UDP-glucose), UDP-galactose and deoxythymidine-diphospho-rhamnose (dTDP-rhamnose) are formed intracellularly. (3) Individual repeating units are attached to an undecaprenol diphosphate anchor (UDA) that is embedded in the cell membrane and the units are synthesised through several GTFs to form. (4) The Wxz flippase protein translocates the repeating sugar units to the outer membrane. (5) The outer membrane protein Wxy polymerises the sugar units into heteropolysaccharides and they are released into the extracellular environment [43,49].

### 1.5. Industrial Applications

The majority of industrially applied polysaccharides are currently derived from plant or algae sources, with Xanthan being the only significant bacterially derived EPS commercially available polysaccharide, constituting 6% of the global EPS market [50]. Bacterial-derived EPS however have some unique strengths over plant or algae-derived ones. Bacterial EPS can be produced from renewable sources and by producing EPS through controlled fermentation a reproducible, high quality and high titer product can be obtained [51]. These qualities have seen the application and integration of EPS in the food industry with preparations used to improve the stability, rheology and texture of many foodstuffs and beverages. Currently, EPS is used in yoghurt, kefir, cheeses, gluten-free products and cereal-based products either in situ or as an ingredient [32,42,52,53]. However, the production of EPS in situ is not always desired in food products, and EPS is well-known for the spoilage of cider and wine [42].

For applications of EPS in situ and as a food ingredient, continuous effort to improve the production process is needed.

### 1.6. Legal Status of EPS as a Novel Food Ingredient

There are, to date, 39 LAB species and 5 Bifidobacteria species on the qualified presumption of safety list (QPS list) issued by the European Food Safety Authority (EFSA) that also have generally regarded as safe (GRAS) status. These are of specific importance because they may facilitate easier application in food matrices [54]. However, there is no EFSA or FDA health claim for the use of EPS from lactic acid bacteria in food products. A few microbial EPS are approved by EFSA and include xanthan gum from *Xanthomonas campestris*, β-glucans from *Saccharomyces cerevisiae* and chitin from *Aspergillus niger* while pullulan from *Aureobasidium pullulans* is currently under investigation to get EFSA approval [55,56,57,58]. As several LAB are approved for food use EPS can be used as an ingredient when produced in situ, but to be used as a purified ingredient in the EU several steps would need to be taken to comply with the EFSA regulations on novel food ingredients [59]. Purified EPS falls within this category of novel food ingredients as it has not traditionally been consumed as a food product and will as such need to be approved as safe for human consumption.

## 2. Production and Process Conditions

When producing EPS from lactic acid bacteria, the primary aim is to optimise the process to achieve the highest yield possible. In comparison with other EPS-producing strains, the yields obtained from LAB are quite low. To obtain an economically feasible production of EPS to use as a food ingredient, it is suggested that yields should be in the range of 10–15 g/L [46].

The selection of an appropriate strain or strain co-culture is the first step for EPS production. The proper cultivation conditions and parameters such as the composition of culture media, the micronutrient profiles and the fermentation setup can thereafter be adjusted to fit the optima of the selected strain. As most lactic acid bacteria grow well on lactose, milk and whey-based media present an alternative to complex media, thereby keeping production costs lower.

### 2.1. Effects of Strain Selection and Carbon Sources

EPS-producing strains are capable of growing on a wide variety of carbon sources including sucrose, glucose, galactose, fructose, lactose, maltose and mannose as well as dairy- and starch-based media [60,61]. The preferential carbon source for optimal EPS production is highly species and strain-dependent, and the quantity of carbon does not only affect the growth but also the EPS production, as some studies indicate that for certain strains an excess of sugars can have an EPS-inducing effect [62,63,64].

Li et al. (2016) reported the ability of *S. thermophilus* to produce EPS in media formulations that differed only in carbon sources (glucose, sucrose, lactose, fructose and galactose). Based on EPS production, *S. thermophilus* displayed a preference for sucrose as a carbon source with an EPS production of 108 mg/L, followed by glucose and lactose which only yielded slightly lower amounts [65]. Similarly, other studies have found the best EPS yields on sucrose and lactose with versions of lactose appearing to be the most utilised carbon source, in either its pure form, milk, reconstituted skim milk, whey or deproteinized whey (DW) (Table 1). Although sucrose in most studies appears to be an effective carbon source, one study found the contrary with a three-times lower yield of EPS of 34 mg/L on sucrose in comparison with lactose (114 mg/L) and glucose (120 mg/L) highlighting the importance of considering the variance between strains [62].

*L. delbrueckii* subsp. *bulgaricus* have been grown in media with glucose, fructose, lactose and mannose as carbon sources and produced the highest yield of EPS when grown on either glucose or lactose, with substantially lower yields when grown on fructose [62,66,67]. This lower yield of fructose was hypothesized to be due to a more complex pathway for the production of EPS [66]. Therefore, most studies focus on growth in glucose, lactose or dairy-based media (Table 1).

Efficient production of EPS has also been reported for *L. casei* [68]. The growth and EPS production for *L. casei* has been investigated on several carbon sources, but there are diverging and contradictory conclusions on which carbon source is best in terms of productivity, which could be due to the strain variance between different research groups. Some studies report the highest yields when utilizing galactose as a carbon source with EPS yields of 120 mg/L [68,69] while another study reports the lowest yields when *L. casei* was grown in a galactose-based medium and the best yields in a glucose-based medium with an EPS-production of 160 mg/L [63,70]. In contrast to other strains that can utilise lactose as a carbon source, *L. casei* does not respond to this when present in the culture media [63,68].

*L. plantarum* has shown the ability to grow and produce EPS production in media containing lactose, glucose, sucrose, fructose and galactose as carbon sources. The highest EPS production yields have been found with glucose-based media with a yield of EPS of 956 mg/L [71]. Although high EPS yield has also been observed in lactose-based media with a yield of 140 mg/L, *L. plantarum* had a lower growth rate on lactose compared to glucose, galactose and sucrose [72].

Polak-Berecka et al. (2014) and Oleksy-Sobczak et al. (2020) used a single-factor experimental approach to determine the optimal carbon source for the growth of *L. rhamnosus.* Experimentation was carried out, creating a modified MRS-based media, where the carbon source (glucose) was iteratively replaced with the following: lactose, galactose, fructose, sucrose or maltose [73,74]. In the study by Polak-Berecka et al. (2014) glucose followed by galactose and lactose resulted in the highest yields of EPS with production values of 185 mg/L, (165 mg/L) and 152.84 mg/L respectively. These substrates were reported to produce four times higher quantities of EPS compared to sucrose, fructose and maltose which were also tested [73]. On the contrary, the study by Oleksy-Sobczak et al. (2020) tested the same carbon sources and found fructose and sucrose to provide similar EPS productivity to that attained from using glucose or lactose [74]. This study also observed a positive influence on the EPS yields when cultivating *L. rhamnosus* strains in a combination of different carbon sources as a further six-to-eight-fold yield was observed. This high production of EPS on glucose and lactose has also been observed in other studies [75,76], with a high yield of EPS observed by Dupont et al. (2000) using MRS as media reaching an EPS yield of 1138 mg/L or MRS with lactose reaching an EPS yield of 1275 mg/L [75]. These findings allude to high variance among strains and the importance of tailoring the carbon composition to the working strain for optimum production yields.

With lactose being a well-documented carbon source, the growth of *L. rhamnosus* has also been optimized in several dairy-based media. Utilising pure unhydrolyzed whey directly as a feedstock in fermentation is challenging due to its low concentration of nitrogen sources which would lead to substrate limitation for microbial growth if additional supplementation of yeast extract or salts is not used [80]. An alternative method to supplement whey is the hydrolysis of the protein. By applying this method a strain of *L. delbrueckii* subsp. *bulgaricus* which did not produce any EPS in unhydrolyzed media was able to produce EPS with yields between 313 and 330 mg/L in hydrolysed whey [80].

Milk alone supports the growth and EPS production of LAB and has been compared to a chemically defined medium with either glucose or lactose. A skim milk powder-based media yielded a higher EPS fraction than chemically defined media, possibly due to the release of peptides from the milk caused by bacterial proteases [85,113]. An increase in EPS production has been observed in milk that has been enhanced with whey proteins, where the EPS yield doubled from 458 mg/L to 1029 mg/L in one study [114] and from 70 mg/L to 330 mg/L in another study [111].

Whey also represents a much more economically feasible feedstock than chemical media, and the comparison of yields between chemical media and whey is therefore of relevance [94]. The EPS production of *L. delbrueckii* subsp. *bulgaricus* in a milk-based medium and a chemically defined medium was similar in both [113]. A basal minimal medium (BMM) was compared to a whey medium with additional yeast extract and BMM salt and amino acid solution for *L. rhamnosus*. Here, a fourfold higher yield of EPS was found in the whey-based medium with a yield of 2767 mg/L compared to 644 mg/L in the BMM [61]. Research on the utilization of waste-based media is essential for driving forward research on EPS production as production costs of media represent a serious challenge and limitation.

### 2.2. Nitrogen Sources

A nitrogen source is required for the growth of LAB which can be supplied from the media by the addition of yeast extract, peptone, tryptone, casitone or beef extract [111,115,116]. This section reviews the suitability of various nitrogen sources in supporting LAB growth and EPS production. Several nitrogen sources support EPS production, however, yeast extract appears to be the most utilised in the literature providing the highest yields [61,111,115]. Yeast extract provides additional advantages compared with other sources, as it contains a sufficient amount of vitamins to support LAB growth [61]. Some studies indicate an EPS production inhibition effect from excess nitrogen in the fermentation broth when using yeast extract concentrations above 56 g/L [107,110,117]. The necessity of the correct nitrogen balance was highlighted in a study by Shene et al. (2008), with a media based on lactose with yeast extract, polypeptone, manganese(II) sulphate and magnesium sulphate Trials with a nitrogen concentration varying by +/− 50% from a base concentration of 25 g/L were conducted. The increase in nitrogen concentration had no effect on EPS yield (68 mg/L) and productivity (12.3 g/L/h), while the lowered concentration of nitrogen resulted in a lower final yield of EPS of 53 g/L and a 50% reduced productivity rate of 6.2 g/L/h [68]. The importance of the carbon/nitrogen ratio has also been demonstrated in another study where the addition of nitrogen had an increasing effect until the initial concentration of total nitrogen solids reached 4.2%, after which total EPS yield would decrease [107].

### 2.3. Amino Acids, Salts, and Vitamins

LAB require a range of amino acids for optimal growth, and omission of the amino acids asparagine, glutamine and threonine can result in reduced growth [82,116]. Amino acids are not directly involved in EPS synthesis, but can still serve as sources of carbon and nitrogen in the growth media [106]. The addition of amino acids to growth media has not been shown to increase EPS yield for either *S. thermophilus* or *L. rhamnosus*, nor has the omission of essential amino acids shown to decrease EPS yield relative to cell density in *L. delbrueckii* subsp. *bulgaricus* [61,82,106]. Macedo et al. (2002) reported that the addition of additional amino acids and salts to a whey and yeast extract-based medium produced high EPS yields of 2775 mg/L [61]. The addition of purified amino acids to a fermentation broth is very expensive for industrial scale-up and not preferred in industrial food processing [61].

The addition of different salts can also increase the growth of LAB and thereby enhance EPS production with some ions speculated to be essential for EPS biosynthesis [116,118]. Mn^2+^ and Mg^2+^ are essential growth factors for lactobacilli, and these two trace elements have been shown to increase EPS yield [119]. Mg^2+^ is an essential mineral as it influences the activity of phosphoglucomutase, an essential enzyme that participates in EPS biosynthesis [61]. An increased presence of Mn^2+^ does not lead to an increased yield of EPS, and Mn^2+^ has proved to be essential only at low concentrations [118]. The addition of a salt solution (sodium acetate, ammonium citrate, KH_2_PO_4_, K_2_HPO_4_, MgS0_4_·7H_2_O, FeSO_4_·7H_2_O and MnSO_4_·2H_2_O) to a whey and yeast extract-based medium significantly increased the EPS yield of *L. rhamnosus* from 438 mg/L to 1673 mg/L [61]. Similar results have been obtained in kefir fermentation where the addition of MnSO_4_·4H2O, MgSO_4_·7H_2_O, FeCL_3_ and KH_2_PO_4_ resulted in an increased EPS yield by *L. kefiranofaciens*, with FeCl_3_ inducing the highest increase [120].

To investigate the influence of specific vitamins on the growth of *L. delbrueckii* sups. *bulgaricus*, a one-at-a-time omission of several vitamins in a growth media was tested. Only riboflavin, calcium pantothenate and nicotic acid appeared to be essential for growth [82]. By omitting several vitamins at once from the media except for the aforementioned vitamins, a decrease in the growth of *L. delbrueckii* sups. *bulgaricus* was found, but with a significant increase in EPS production [82]. Another study did not find an increased EPS yield by vitamin addition for *L. rhamnosus* in a media containing whey and yeast extract, potentially due to a sufficient amount of vitamins already present in the yeast extract [61]. Similar results were obtained for kefir grains grown in milk, which also did not show an increase in growth by vitamin addition [120]. The specific addition of vitamins to a growth media can be expensive when producing EPS on a larger scale, and the little evidence of a stimulatory effect makes it non-essential.

### 2.4. Temperature and pH

EPS production is highly dependent on the culture conditions pH and temperature [114]. The specific dependency of both pH and temperature is additionally highly strain-dependent. Studies have found EPS production to be highest at a pH between 5 and 7 [88,93,114,121]. A higher EPS yield has moreover been found to be connected to the presence of pH control as opposed to acidified batches [82,94]. One study was able to produce 1029 mg/L of EPS from *S. thermophilus* at a constant pH of 5.5 after 24 h but yielded 491 mg/L in a similar fermentation without pH control [114].

EPS yield has generally been higher when strains were grown in sub-optimal temperatures for bacterial growth with decreases in yield observed when grown in over-optimal temperatures [77,83]. In *L. lactis*, *L rhamnosus* and *L. plantarum* the optimum temperature range for EPS production is between 18 and 25 °C [64,70,72]. This range is slightly higher for *S. thermophilus* with optimum temperatures reported between 32 and 42 °C, [104,109,114,122].

An important way of increasing the yield of EPS produced in fermentation is by inhibiting EPS degradation. Degradation can be caused due to enzymatic activity, and pH and temperature are important factors that can influence this degradation [104,106]. Studies have been conducted in which enzyme activity has been stopped at the end of fermentation through a temperature step with an increase from 42°C to 90°C or an acidification step from a pH of 6 to 3 of the broth. In the study, pH and temperature shifts lead to no observable EPS degradation over time, whereas control experiments with temperature and pH maintained at 42 °C and 6.2, respectively, indicated a clear decrease in EPS [106]. This study indicates that stopping enzyme activity at the end of fermentation is a useful method to preserve EPS yields.

### 2.5. Fermentation Technologies

Batch procedures have proved suitable for industrial EPS production, while batch processes and shake flasks are widely employed for media optimisation (Table 1), the highest production yields have been observed with continuous cultures or fed-batch cultures [81,91,92,101,123].

Repeated batches and continuous cultures with *L. rhamnosus* have been attempted with high yields and productivities [91,92]. In supplemented whey-based media, the productivity of EPS was doubled from 110 mg/L/h after 18 h in a simple batch to 250 mg/L/h for repeated immobilized cell culture after 7 h [91]. The technique of immobilized cell culture was continued in a later study followed by a phase of continuous fermentation with free cells for a total of 32 days. This fermentation setup resulted in larger aggregates of both biomass and EPS, and at the maximum productivity reached at 24 days, they estimated volumetric productivity of 15.8 g/L/h for biomass and 2240 mg/L/h for EPS [92].

The co-cultivation of the kefiran-producing species *L. kefiranofaciens* and *S. cerevisiae* in a fed-batch fermentation set-up has been performed in two studies [103,124]. A batch reactor set-up with a media consisting of MRS with lactose saw an accumulation of lactate that inhibited polysaccharide production. By switching to a fed-batch setup with a lactose feed, the yield was increased from 4.5 g/L in batch to 6.3 g/L in fed-batch. A high yield of EPS was found in another co-culture of *L. kefiranofaciens* and *S. cerevisiae* also grown in a fed-batch set up with MRSL media, but here with a feed consisting of 150 g of lactose and 25 g of yeast extract added at 46 and 64 h [101]. This fermentation yielded a final EPS yield of 5.4 g/L and productivity of 62 mg/L/h. This high yield was associated with a high initial level of carbohydrate in the medium as well as an increase of yeast extract from the feed which thereby leads to a beneficial change in the carbon/nitrogen ratio [101].

### 2.6. The Effect of Strain Interactions on EPS Production

There are only a few studies that have investigated the relationship between strain interactions and EPS production. Sizu et al. (2003), investigated the performance of a co-culture including a non-EPS-producing strain and an EPS-producing strain of *S. thermophilus* at different ratios to produce EPS. Here, the optimal ratio was found to be in a co-culture consisting of 75% *S. thermophilus* and 25% non-EPS *S. thermophilus*, which yielded a final EPS concentration of 832 mg/L compared to 458 mg/L in a pure culture of EPS-producing *S. thermophilus*. This indicates a synergistic relationship between the two *S. thermophilus* strains [114]. The classic yoghurt starter cultures *L. delbrueckii* subsp. *bulgaricus* and *S. thermophilus* have also been grown in co-cultures with an observed higher yield compared to those grown in monocultures [105,112]. In addition to growing the two yoghurt cultures together, the EPS-producing lactose-negative yeast *Rhodotorula rubra* was also added, which yielded an even higher amount of EPS of 19.3 g/L [112]. Another documented bacteria–yeast co-culture consisting of *L. kefiranofaciens* and *S. cerevisiae* has also resulted in high EPS yields of 5.4 g/L [101,124].

These higher yields in co-cultures can be assumed to be due to the stimulation of yeast growth through lactic acid production by the LAB which can act as a substrate for the yeast as well as improve the growth environment by lowering the pH. The yeast will produce metabolic by-products such as vitamins and amino acids provided that will in turn then stimulate the growth of the LAB [101,112]. Furthermore, it has been proposed that the mannan present in the cell walls of *S. cerevisiae* could have a stimulatory effect on EPS production from *L. rhamnosus.* The high-producing strain *L. rhamnosus* RW-9595M was able to produce 42% more EPS (1350 mg/L) when grown in a co-culture with the yeast *S. cerevisiae* [98].

### 2.7. Production Challenges

During the production of EPS, the viscosity of the fermentation broth will change as the concentration of polysaccharides increases. In the early stages, the broth displays Newtonian fluid behaviour but will later become a highly viscous non-Newtonian fluid with shear-thinning behaviour [125,126]. If yeast is used to cultivate the fermentation, the growth of the cells alone may also cause an increase in viscosity [127]. The initial bioreactor growth will therefore have a viscosity that is easy to mix due to its homogenous nature. In its early stages, the process will require low energy inputs while at later stages, it will become more difficult to maintain proper mixing and aeration [128]. Whether oxygen and aeration will need to be supplied to the process will be dependent on the use of either aerobic, microaerophilic or anaerobic cultures. The increasing viscosity of the culture can make it difficult to cultivate aerobic cultures as the efficiency of the oxygen transfer rate is reduced [127]. The shear-thinning behaviour in an EPS-producing culture can also lead to inconsistencies in the broth with a low viscosity near the impeller and a higher viscosity further away which in turn can lead to misrepresentation of the culture when sampling [127]. Other consequences of this broth heterogeneity can be improper mixing of titrants for pH control or heat transfer limitations. This heterogeneity in the broth will only increase with the scale-up operation of the bioreactor as a result of the bigger physical distance between the impeller and vessel walls [129].

## 3. Extraction and Purification of Exopolysaccharides

Equally as important to the variables that influence the successful cultivation of an EPS-producing LAB are the methods which allow for the successful isolation, recovery and quantification of polysaccharide produced. There are several suggested approaches which are highly dependent on the type of media used in the production steps as well as the final desired usage of the EPS product.

### 3.1. Isolation and Recovery

**Figure 8:**
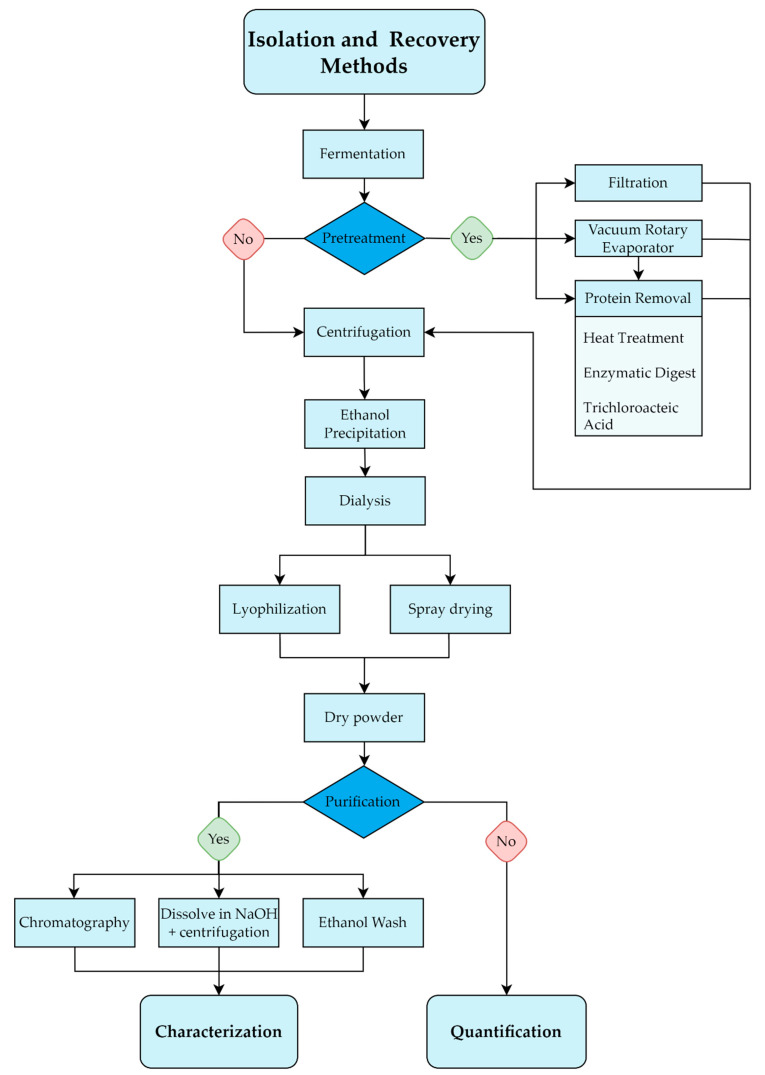
Diagram of isolation and recovery of EPS from the end of fermentation to either characterisation or quantification.

Recovery of EPS from culture is usually achieved in three steps: (1) removal of cells and other undesirable compounds, followed by (2) precipitation of the polymer in the cell-free supernatant, before (3) drying the precipitated polymer (Figure 8) [130].

#### 3.1.1. Pre-Treatments

The simplest method for recovery and isolation can be found in experiments applying a defined media, a centrifugation step for cell removal and isolation by simple dialysis with water [131]. Recovery from liquid media is highly dependent on the usage of either complex or defined media, and it might be necessary to employ some pre-treatment steps for the inactivation of certain enzymes or the removal of particular proteins [132]. In many instances, especially when applying a complex media, a heating step of 90–100 °C is used to kill the microorganisms and inactivate enzymes that might be present [61,73,76,133]. Furthermore, the effect of heat treatment on final EPS yield before isolation has been evaluated. It was concluded that a heat treatment step was critical for the complete recovery of EPS resulting in significantly higher yields [134]. A vacuum rotary evaporator has been employed to concentrate the fermentation broth before EPS isolation; however, this method is seldom reported [73,95].

For a thorough removal of the protein fraction, a precipitation step is usually employed either by the addition of trichloroacetic acid (TCA) in concentrations varying between 4 and 14% under mixed conditions or via enzymatic digestion by proteases or a combination of both [132,135]. This precipitation step is generally applied before EPS is removed from the media [65,136]. Whether a TCA step is necessary, depends on the final objective: if the goal is quantification it can be omitted whereas it would be needed for specific characterization of the polysaccharide structure [135]. As an alternative to TCA and ethanol treatments, ultrafiltration has been suggested as a faster and more accurate method for EPS isolation [94]. Subsequent removal of the cell fraction and coagulated proteins from the liquid can be obtained by centrifugation [23,76] which can be followed by a microfiltration or ultrafiltration step for further purification [89,110].

#### 3.1.2. Precipitation and Dialysis

With the pre-treatments applied, the next step is precipitation which is most commonly done with ethanol (Table 2). Rimada and Abraham (2003) compared different methodologies for the isolation of EPS: one or two steps of ethanol precipitation, one-step ethanol precipitation followed by dialysis, dialysis with membranes of different cut-offs (6000, 8000, 12,000, and 14,000) and TCA precipitation. The single-step alone with ethanol was not adequate for isolation as residual lactose was found to co-precipitate with EPS, resulting in false high values. Two-step ethanol, ethanol precipitation and dialysis with membranes with molecular weight cut-off lower than 8000 all yielded similar results and were deemed suitable methods for EPS isolation. A low result from TCA precipitation was obtained, and it was speculated that this was falsely negative because treatment with TCA leads to a very thorough removal of proteins and impurities that can also result in EPS precipitation, leading to a loss of final polymer concentration of up to 50%. It is therefore recommended to wash the TCA precipitate at least once. The temperature of ethanol varies between studies, but a comparison of two ethanol temperature points of −20 °C and 4 °C showed no significant difference in final EPS [134]. In a few cases, acetone or a combination of ethanol and acetone has been applied [104,106,112]. Dialysis is applied as the last step before drying and is an essential step since it is used for the removal of carbohydrates with low molecular mass [132].

#### 3.1.3. Drying and Characterisation

The EPS isolation is usually finalised by lyophilization, spray-drying or spray-freeze drying to a powder [135,137]. If the goal is specific EPS characterization and not quantification, the EPS lyophilizate can be purified by washing the powder with ethanol [138] or by dissolving it in sodium hydroxide followed by centrifugation to further eliminate contaminants [139]. In addition, both ion-exchange chromatography [99,140] and size exclusion chromatography [23,89,141] are applied in studies that aim to characterise the EPS produced by LAB. To examine the structure of EPS, more in-depth methods such as nuclear magnetic resonance (NMR), Fourier transform infrared (FT-IR), scanning electron microscopy (SEM) and transmission electron microscopy (TEM) can be applied [142,143,144,145].

### 3.2. Quantification Methods

**Figure 9:**
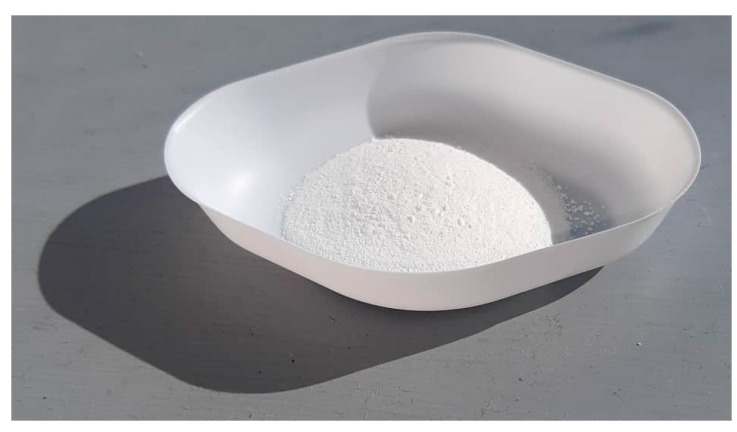
Dextran from *Leuconostoc mesenteroides*, Megazyme (Ireland).

Following the initial steps of isolation and recovery of EPS, a powdered product is obtained (Figure 9). The simplest method for quantification is weighing the powder, but this method is imprecise as the final dry weight will include any impurities present [104,146]. The method used most frequently is a measurement of the carbohydrate contents by the colourimetric phenol-sulfuric acid method, first proposed by Dubois et al. (1956). Simple sugars, oligosaccharides, polysaccharides and their derivatives will obtain an orange-yellow colour when being treated with phenol and sulfuric acid [147]. The presence of contaminating carbohydrates remaining present in the fermentation broth can however also make this quantification method imprecise [148]. Another colourimetric approach to EPS quantification is the usage of the anthrone reagent [103,149], which is mostly applied for strains with higher productivity as it can only be considered accurate on yields above 10 mg/L [108,134].

**Figure 10:**
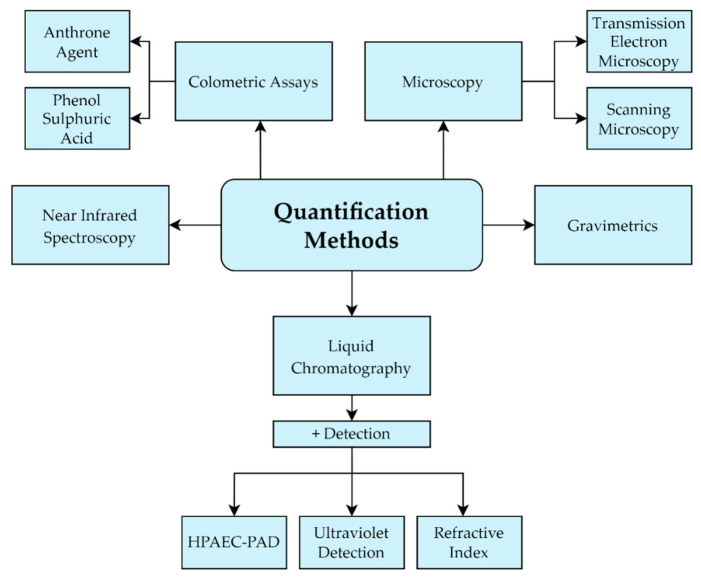
Overview of quantification methods.

Other methods for EPS quantification include the use of liquid chromatography such as separation by high-performance liquid chromatography (HPLC) combined with detection by refractive index (RI), anion exchange chromatography (AEC) or high-performance anion-exchange chromatography pulse amperometric detection (HPAEC-PAD) [96,108,150]. The use of HPLC can furthermore be combined with ultraviolet detection to account for the presence of proteins [138]. Near-infrared spectroscopy (NIRS) has also been used for simultaneous quantification of lactic acid, lactose and EPS directly in liquid samples from a fermentation. The yields were measured both through NIRS and conventional HPLC for lactic acid and lactose, while ultrafiltration and phenol-sulfuric method was utilized for EPS. These were then compared and showed coefficient of relation values of 99% for lactic acid and lactose and 91% for EPS, suggesting NIRS as a rapid tool for monitoring EPS in fermentations [151]. Other direct methods for EPS monitoring include SEM and TEM [99,139]. The different methods for quantification are outlined in Figure 10.

Due to the large amount of purification and quantification methods of EPS, the yield can be difficult to compare between studies, as some techniques can result in considerable different EPS measures [92].

## 4. Health Benefits of Exopolysaccharides

EPS have a wide range of described biological functions due to their large structural diversity which is based on strain selection and fermentation conditions.

**Figure 11:**
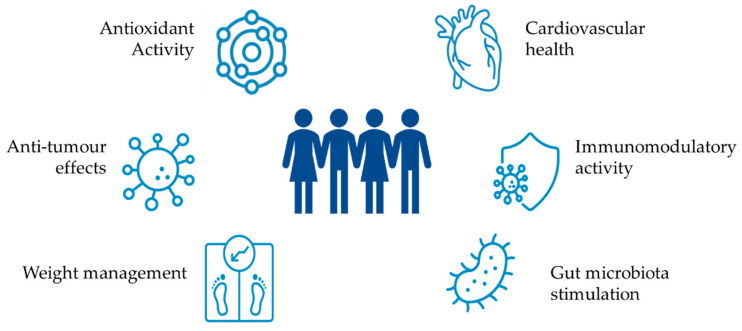
Health benefits associated with exopolysaccharides.

The postbiotic effect exerted by EPS includes antioxidant effects, immunomodulatory activities, anti-tumour effects, gut microbiota stimulation and cholesterol-lowering activities [35,36]. The EPS produced by lactic acid bacteria has received substantial attention for its various therapeutic applications which are discussed in this review and displayed in Figure 11.

### 4.1. Gut Microbiota Stimulation

Some studies indicate that EPS demonstrates the potential to stimulate and enhance the populations of beneficial bacteria in the gut [152].

To act as a stimulant in the gut it is of great importance that the EPS can survive the harsh environment in the gastrointestinal system. Some literature report that strains of *S. thermophilus* does not produce EPS that possess this ability, while there are reports of the EPS produced by the strains *Lactococcus* and *Lactobacillus* maintaining their integrity through digestive stress, consequently making the potential of the polysaccharide strain-dependent [24,153].

EPS isolated from strains of *L. sanfranciscensis*, *L. rhamnosus* and *L. plantarum* have all been shown to exercise bifidogenic effects [95,154,155]. This implies that they show a stimulatory effect on species within Bifidobacteria that are well known for their beneficial influence on the health of the gastrointestinal tract [156]. EPS has been shown to not only stimulate Bifidobacteria but also have a more non-specific stimulatory effect on lactobacilli, enterococci, *Prevotella* and *Bacteroides* [157]. Simultaneously, it has been observed that EPS did not stimulate the growth of pathogens from *Clostridium* clusters. The genus *Candida* normally consists of commensal species inhabiting the gut, but some species can shift to become opportunistic pathogens. The presence of probiotic bacteria is known to inhibit the growth of pathogenic *Candida* species, and some evidence suggests that the EPS layer might aid in this inhibition [158,159]. The *Candida* inhibiting effect of *L. rhamnosus* was tested by comparing the effects of *L. rhamnosus GG*, an EPS-deficient mutant and purified EPS from *L. rhamnosus GG* and the inhibitory effect on hyphal formation and adhesion of *Candida* was suggested to be a result of EPS interference [159]

The stimulation of healthy gut bacteria by ingestion of EPS is decisively well characterized in vitro, but more evidence of this effect also occurring in vivo is still necessary.

### 4.2. Immunomodulatory Activity

Studies have indicated EPS may exert immunomodulatory effects, those of which may be dependent on strain and in turn affect the physical properties of the polysaccharides produced. EPS have been seen to act as stimulators on immune cells when they have a phosphate present in their composition, giving them a negative charge [160]. Furthermore, EPS structure seems to play a role, small EPS molecules can stimulate immune cells. Larger EPS strands have been shown to elicit an immune-suppressive response [161].

The stimulating effect of a phosphate-containing EPS from *L. lactis* subsp. *cremoris* was demonstrated in a mouse spleen macrophage, where EPS induced synthesis of the cytokines interferon-gamma (IFN-γ) and interleukin-1 (IL-1α) [162]. The same group also researched an immunostimulatory effect in a study aiming to produce charged and uncharged EPS with strains of *L. delbrueckii* subsp. *bulgaricus*. Only the charged EPS was able to stimulate immune cell proliferation [163]. Another study made similar observations that separated high-molecular-weight EPS from neutral EPS produced by a strain of *L. delbrueckii* subsp. *bulgaricus* and observed a significantly higher concentration of INF-γ produced by mouse splenocytes in the charged EPS fraction [164]. This effect did not seem to be dose-dependent, as the stimulatory effect was obtained in similar quantities in concentrations varying between 20 and 500 µg/mL. EPS was also administered to mice both directly and through yoghurt fermented with *L. delbrueckii* subsp. *bulgaricus*. A dose-dependent increase in natural killer cell activity was observed in mice fed EPS directly with the best response at a maximum value of 30 mg/kg together with a slight increase in INF-γ and a slight decrease in IL-4. Similar results were obtained for the yoghurt-fed mice [164].

The stimulation of cytokine production by EPS in macrophages has been demonstrated especially for the stimulation of tumour-necrosis factor α (TNF-α), IL-6 and IL-1β [165,166]. Kefiran has similarly shown cytokine stimulatory effect, with EPS produced from *L. kefiranofaciens* administered to mice resulting in increases of IL-6, IL-10, and IL-12 in the small intestine and increases in serum levels of IL-4, IL-6, IL-10 and IFN-α [167,168].

Conversely, another study found an immunosuppressive and anti-inflammatory effect of the EPS produced by the isogenic strain of *L. rhamnosus* utilized by Chabot et al. (2001) [166,169]. This study compared the parental strain of *L. rhamnosus* ATCC-9595 to an isogenic variant, *L. rhamnosus* RW-9595M and saw the same pro-inflammatory stimulation by ATCC-9595 that was previously observed with increases of TNF-α, IL-6 and IL-12. RW-9595M on the other hand induced high levels of IL-10 with almost no TNF-α, IL-6 and IL-12. Hydrolysis of EPS resulted in a change in molecular weight, but not in the electronic charge and also affected cytokine production, indicating a mass-dependent effect of EPS on cytokine induction signalling pathways [169]. This immune stimulating or immunosuppressive mass-dependent effect was also suggested in a study using EPS-producing Bifidobacteria strains. Here, it was concluded that EPS with high molecular weight could generally diminish the immune system, while smaller EPS would elicit an increased response [170].

### 4.3. Antioxidant Activity

EPS produced from LAB have been found to have antioxidant activity and presents an alternative to synthetic antioxidants that can also possess cytotoxic and carcinogenic activity [171,172]. Removal of high levels of reactive oxygen species is necessary to control oxidative stress [173].

In vitro studies on the EPS produced by *Bacillus coagulans*, *Weissella cibaria*, *L. plantarum* and *L. paracasei* have shown that the polysaccharides produced can have antioxidant and free radical scavenging activity [165,174,175,176]. This antioxidant effect has also been studied in a hyperlipidaemic rat model with EPS from a strain of *L. casei* and a senescent mouse model suffering from oxidative stress with EPS from *L. plantarum* [177,178]. Both EPS from *L. casei* and *L. plantarum* demonstrated an antioxidant effect with an increase of activity in enzymes important for the antioxidant defence system of the cell (superoxide dismutase and glutathione peroxidase) while also reducing levels of malondialdehyde, a marker for oxidative stress [177,178].

### 4.4. Anti-Tumour Activity

Studies conducted on the anti-tumour activity of EPS are very early stage, but there are still indications that EPS indeed could be used as an addition to current cancer treatments [179]. The administration of 10 mg/mL EPS from a strain of *L. acidophilus* has been shown to inhibit cell proliferation of colon cancer cells while being non-toxic to normal colon cells [180,181]. The same anti-proliferative effect on cancer cells has also been observed on EPS fractions doses of 10 mg/mL from *L. plantarum* and *L. helveticus* on breast cancer and gastric cancer cell lines, respectively [182,183]. A lower dose of EPS from *L. plantarum* demonstrated moderately effectivity at 5 mg/mL against hepatic cancer cell lines and was significantly effective at 600 µg/L against colon and gastric cancer cells [184]. A recent study showed that concentrations of 400 µg/L of lyophilized EPS from the lactobacilli *L. plantarum*, *L. rhamnosus*, *L. brevis* and *L. delbrueckii* subsp. *bulgaricus* induced 40% apoptosis and decreased the viability of colon cancer cells [2].

### 4.5. Cardiovascular Health

EPS can aid in the improvement of cardiovascular health as it possesses cholesterol-binding abilities [43]. It is not clear, however, in the majority of research examining the effect of EPS as a hypocholesterolemic agent as to whether the effects are attributed to the EPS specifically, or if the bioactivity is stemming from the entire EPS-producing organism. Milk fermented with either an EPS-producing or non-EPS-producing strain of *L. lactis* subsp. *cremoris* were fed to rats on high-cholesterol diets. Significant reductions in serum cholesterol and a significant increase in the high-density lipoprotein/total cholesterol ratio were observed for rats fed with milk fermented by the EPS-producing strain. It was concluded that this outcome was due to the dietary fibre action caused by the polysaccharide [28]. An in vitro study by Tok and Aslim investigated cholesterol removal by *L. delbrueckii* subsp. *bulgaricus* and observed a maximum cholesterol removal of 31% in the strain that also had the highest EPS production. Furthermore, they observed that cholesterol removal was similar for resting and dead cells, suggesting that this removal could indeed be due to components like EPS synthesized before cell death, rather than an effect caused by the living cell itself [185]. The EPS produced by *L. kefiranofaciens* significantly suppressed increases in blood pressure and reduced serum cholesterol for hypertensive stroke-prone rats that were fed diets high in cholesterol [186]. These results are further supported by the cholesterol-lowering effects observed in *L. paracasei*. Dietary administration of *Lactobacillus mucosae* and *L. paracasei* reduced total serum and liver cholesterol and serum triglycerides in mice fed a high-fat diet compared to the control group that were administered non-EPS producing *L. paracasei* or a placebo [3].

Drugs that inhibit the Angiotensin-converting enzyme (ACE) are common for the treatment of hypertension. ACE is needed for the conversion of angiotensin 1 to angiotensin 2 which narrows the blood vessels and can cause higher blood pressure [187]. The antihypertensive effects of EPS from *L. casei* were studied in an in vivo model and found a significant reduction in the systolic blood pressure with no effect on the heart rate. This effect was determined to be due to inhibition of the angiotensin-converting enzyme [188].

### 4.6. Weight Management

Studies have indicated EPS may also have application as an anti-obesity treatment [141,189]. The anti-obesity effect of EPS from *L. rhamnosus* was observed in a mouse model [141]. Mice on a high-fat diet that were injected with EPS at a concentration of 50 mg/kg every two days for two weeks. These mice had significantly reduced fat pads with much smaller adipocytes. This anti-obesity effect was similarly observed for high-fat diet mice fed with diets containing 5% EPS isolated from kefir grains [190]. Here, researchers observed a reduction in body weight gain, adipose tissue weight and low-density lipoprotein cholesterol. This study also showed an increase of species in the genus *Akkermansia*, which have shown to be inversely associated with obesity [191].

EPS was tested for its anti-obesity effect by comparing the effect of the addition of either a high EPS-producing strain of *L. casei* or a low EPS-producing strain of *L. plantarum* to a mouse model with diet-induced obesity. Here, it was concluded that the inclusion of *L. casei* in the diet had a positive impact on the metabolic outcome with a reduction in hepatic triglycerides and cholesterol as well as fat pad weight [192].

Short-chain fatty acids (SCFA) have been described as having effects on body composition and reduction in body weight. In a human gut microbiota model inoculated with faeces from 12 healthy adults, the effect of EPS on the gut microbiota was investigated. An increase in the production of SCFAs has been observed in the fermentations of inoculated EPS in comparison to the controls [189]. Short-chain fatty acids have been shown to have effects on body composition and result in a reduction of body weight, leading it to be an important tool when designing foods for weight management [193].

## 5. Conclusions

EPS produced by lactic acid bacteria present the potential for the continuously growing functional food market as an ingredient that can add both value and innovation.

To achieve a high yield of EPS from lactic acid bacteria on an industrial scale, it is important to optimize factors related to EPS productivity such as choice of strain, media selection and bioprocess setup.

Producing EPS as a functional food ingredient is however not without challenges. Due to the nature of the polysaccharide, the fermentation broth will over time become a non-newtonian viscous fluid. This presents challenges when ensuring adequate aeration and mixing. In addition, yields obtained by lactic acid bacteria alone remain relatively low and further research into process optimization is required for effective and economic scale-up. The usage of continuous and fed-batch fermentation technology presents an interesting possibility to meet these production challenges.

The composition of the fermentation media is ultimately the deciding factor on which pre-treatment, purification, and extraction methods to apply. The appropriate method to apply will also depend on whether the goal is to characterize the specific EPS structure or quantify the fraction. The quantification methods also differ, ranging from colourimetry and column separation to spectroscopy and direct visualization through electron microscopy.

EPS show great promise as a diverse therapeutic agent and has been to have immunostimulatory effects, stimulate healthy gut bacteria, possess antioxidant and anti-cancer activity as well as work as an aid for better cardiovascular health and weight management. Currently, most studies are however limited to showing an effect in vitro and more in vivo intervention studies will need to be performed to confirm the health benefits of EPS. With better substantiation of the health benefits conferred from EPS, it will be important to get an EFSA health claim in order to utilize it as a purified ingredient in food products.

## Figures and Tables

**Table 1:** Summary of EPS-producing strains and yields.

Stain	Media	Fermentation	Yield	Ref
*L. casei*	BMM w. glucose	Flask	160 mg/L	[63]
*L. casei*	Skim milk	Flask	600 mg/L	[70]
*L. casei*	Galactose + tryptone + MnSO_4_ + CaCl_2_	Flask	120 mg/L	[69]
*L. casei*	APTg broth + Ca^2+^ + Mn^2+^	Flask	124 mg/L	[68]
*L. casei*	Galactose + tryptone + MnSO_4_ + CaCl_2_	Flask	488 mg/L	[77]
*L. casei*	Skim milk + APTg broth	Flask	120 mg/L	[78]
*L. casei*	Skim milk	Flask	121 mg/L	[79]
*L. delbrueckii* subsp. *bulgaricus*	MRS + glucose	Flask	255 mg/L	[62]
*L. delbrueckii* subsp. *bulgaricus*	Hydrolysed whey	Single batch—free cells	325 mg/L	[80]
*L. delbrueckii* subsp. *bulgaricus*	Whey + lactose + KH_2_PO_4_ + NH_4_Cl + casamino acids	Continuous fermenter	2.13 g/L	[81]
*L. delbrueckii* subsp. *bulgaricus*	Defined medium	Single batch—free cells	250 mg/L	[82]
*L. delbrueckii* subsp. *bulgaricus*	Defined medium w. glucose	Flask	36.8 mg/L	[83]
*L. delbrueckii* subsp. *bulgaricus*	Defined medium w. fructose + glucose	Flask	80 mg/L	[66]
*L. delbrueckii* subsp. *bulgaricus*	Semi-defined media	Flask	220 mg/L	[84]
*L. delbrueckii* subsp. *bulgaricus*	Milk Chemically defined medium	Flask Flask	170 mg/L 174 mg/L	[85]
*L. delbrueckii* subsp. *bulgaricus*	Lactose (from whey) + YE + peptone + tween80 + MgSO_4_ + MnSO_4_	Continuous culture	830 mg/L	[86]
*L. delbrueckii subsp.* *bulgaricus* *S. thermophilus*	Reconstituted milk	Flask	240 mg/L	[87]
*L. helveticus*	Skim milk	Single batch—free cells	549 mg/L	[88]
*L. helveticus*	Whey + lactose + peptone + MnSO_4_	Flask	658 mg/L	[89]
*L. lactis* subsp. *cremoris*	Defined media	Single batch—free cells	520 mg/L	[90]
*L. rhamnosus*	Whey + YE + tween 80 + MgSO_4_+ MnSO_4_	Single batch—free cells Repeated-batch cultures—ICT	2.3 g/L (110 mg/L/h) 1.7 g/L (250 mg/L/h)	[91]
*L. rhamnosus*	Whey + YE + tween 80 + MgSO_4_ + MnSO_4_	Continuous—ICT	1.8 g/L (542.6 mg/L/h)	[92]
*L. rhamnosus*	BMM w. mannose BMM w. fructose + glucose	Flask	132 mg/L 111 mg/L	[64]
*L. rhamnosus*	CDM	Single batch—free cells	251 mg/L	[93]
*L. rhamnosus*	Whey + yeast nitrogen base	Single batch—free cells	477 mg/L	[94]
*L. rhamnosus*	Whey + YE + salts + AA	Single batch—free cells	2767 mg/L	[61]
*L. rhamnosus*	BMM w. lactose	Single batch—free cells	1275 mg/L	[75]
*L. rhamnosus*	Fructose + glucose + sucrose + K_2_HPO_4_, CH_3_COONa, C_6_H_14_N_2_O_7_, MgSO_4_ + MnSO_4_	Flask	987 mg/L	[74]
*L. rhamnosus*	Fructose + glucose + sucrose + YE + K_2_HPO_4_, CH_3_COONa, + C_6_H_14_N_2_O_7_, MgSO_4_ + MnSO_4_	Flask	900 mg/L	[74]
*L. rhamnosus*	Fructose + glucose + sucrose + K_2_HPO_4_, CH_3_COONa, C_6_H_14_N_2_O_7_, MgSO_4_ + MnSO_4_ + tween	Flask	1138.2 mg/L	[74]
*L. rhamnosus*	MRS w. galactose + YE	Single batch—free cells	210 mg/L	[73]
*L. rhamnosus*	MRS w. lactose	Single batch—free cells	219 mg/L	[95]
*L. rhamnosus*	BMM	Single batch—free cells	495 mg/L	[76]
*L. rhamnosus*	MRS + H_2_O_2_ + CaCl_2_	Static flask	2498 mg/L	[96]
*L. rhamnosus*	Skim milk + sucrose + YNB	Single batch—free cells	256 mg/L	[97]
*L. rhamnosus* *S. cerevisiae*	Whey + YE + corn steep liquor + tween 80 + MgSO_4_ + MnSO_4_	Singe batch—free cells	1350 mg/L	[98]
*L. paracasei*	BMM w. lactose	Single batch—free cells	85 mg/L	[75]
*L. plantarum*	CDM w. lactose	Static flask	140.2 mg/L	[72]
*L. plantarum*	Semi-defined media	Flask	58.7 mg/L	[99]
*L. plantarum*	Glucose + YE + NH_3_SO_4_	Flask	956 mg/L	[71]
*L. kefiranofaciens*	Whey + lactose + glucose + tryptone + sodium acetate + tween 80 + cysteine monohydrochloride	Flask	1215 mg/L	[100]
*L. kefiranofaciens* *S. cerevisiae*	MRS w. lactose	Fed-batch	5.4 g/L	[101]
*L. kefiranofaciens +* *S. cerevisiae*	MRS w. whey lactose	Single batch—free cells Fed-batch	2580 mg/L 3260 mg/L	[102]
*L. kefiranofaciens +* *S. cerevisiae*	MRS w. lactose	Batch Fed-batch	4.5 g/L 6.3 g/L	[103]
*S. thermophilus*	Milk + peptone + YE	Single batch—free cells	166 mg/L	[104]
*S. thermophilus*	Milk + peptone + YE	Flask	284 mg/L	[105]
*S. thermophilus*	Lactose + arginine	Single batch—free cells	1158 mg/L	[106]
*S. thermophilus*	MRS w. lactose and 4.2% nitrogen	Single batch—free cells	1142 mg/L	[107]
*S. thermophilus*	Semi-defined medium	Single batch—free cells	325 mg/L	[108]
*S. thermophilus*	Sucrose + soy peptone	Flask	250 mg/L	[65]
*S. thermophilus*	Whey + YE + tryptone	Single batch—free cells	147 mg/L	[109]
*S. thermophilus*	Lactose from DW + YE + peptone + tween80 + MgSO_4_ + MnSO_4_	Single batch—free cells	106 mg/L	[110]
*S. thermophilus*	Milk + tryptone	Single batch—free cells	507 m/L	[111]
*S. thermophilus +* *L. delbrueckii* subsp. *bulgaricus +* *R. rubra*	Whey + (NH_4_)_2_SO_4_ + KH_2_PO_4_ + MgSO_4_ + YE	Single batch—free cells	19.3 g/L	[112]

**Table 2:** Purification and quantification methods of EPS produced by LAB.

Microorganism	Precipitation	Protein Removal	Other Treatment	Quantification	Ref
*L. casei*	Ethanol	TCA	-	Phenol/sulphuric acid	[63]
*L. casei*	Ethanol	Pronase digestion	Ultrafiltration	Phenol/sulphuric acid	[70]
*L. casei*	Ethanol	-	-	Phenol/sulphuric acid	[69]
*L. casei*	Ethanol	Pronase digestion	-	Phenol/sulphuric acid	[68]
*L. casei*	Ethanol	-	-	Phenol/sulphuric acid	[77]
*L. casei*	Ethanol	Pronase digestion	-	Phenol/sulphuric acid	[78]
*L. casei*	Ethanol	Pronase digestion	-	Phenol/sulphuric acid	[79]
*L. delbrueckii* subsp. *bulgaricus*	Ethanol	TCA	-	Phenol/sulphuric acid	[62]
*L. delbrueckii* subsp. *bulgaricus*	-	Pronase digestion and TCA	-	Phenol/sulphuric acid	[80]
*L. delbrueckii* subsp. *bulgaricus*	Ethanol	-	-	Dry weight	[81]
*L. delbrueckii* subsp. *bulgaricus*	Ethanol	TCA	-	Phenol/sulphuric acid	[82]
*L. delbrueckii* subsp. *bulgaricus*	Ethanol	TCA	-	Phenol/sulphuric acid	[83]
*L. delbrueckii* subsp. *bulgaricus*	Ethanol			Phenol/sulphuric acid	[66]
*L. delbrueckii* subsp. *bulgaricus*	-	Pronase digestion and TCA	-	Phenol/sulphuric acid	[84]
*L. delbrueckii* subsp. *bulgaricus*	Ethanol			Phenol/sulphuric acid	[85]
*L. delbrueckii* subsp. *bulgaricus*	Ethanol	TCA	-	Phenol/sulphuric acid	[86]
*L. delbrueckii* subsp. *Bulgaricus +* *S. thermophilus*	Ethanol	Pronase digestion	Ultrafiltration	Phenol/sulphuric acid	[87]
*L. helveticus*	Ethanol	Pronase		Phenol/sulphuric acid	[88]
*L. helveticus*	Ethanol	TCA	Microfiltration	Phenol/sulphuric acid	[89]
*L. lactis* subsp. *cremoris*	-	-	Dialysis	Phenol/sulphuric acid	[131]
*L. lactis* subsp. *cremoris*	-	-	Microfiltration	Gel permeation chromatography	[90]
*L. rhamnosus*	-	-	Ultrafiltration	Phenol/sulphuric acid	[91]
*L. rhamnosus*	-	-	Ultrafiltration	Phenol/sulphuric acid	[92]
*L. rhamnosus*	Ethanol	-	-	Phenol/sulphuric acid	[64]
*L. rhamnosus*	Ethanol	-	-	Phenol/sulphuric acid	[93]
*L. rhamnosus*	-	-	Ultrafiltration	Phenol/sulphuric acid	[72]
*L. rhamnosus*	Ethanol	TCA	-	Phenol/sulphuric acid	[94]
*L. rhamnosus*	-	-	Ultrafiltration	Phenol/sulphuric acid	[61]
*L. rhamnosus*	Ethanol	-	-	Phenol/sulphuric acid	[75]
*L. rhamnosus*	Ethanol	TCA	-	Phenol/sulphuric acid	[74]
*L. rhamnosus*	Ethanol	TCA	Vacuum rotary evaporator	Phenol/sulphuric acid	[73]
*L. rhamnosus*	Ethanol	TCA	Vacuum rotary evaporator	Phenol/sulphuric acid	[95]
*L. rhamnosus*	Ethanol	Heat treatment	-	Phenol/sulphuric acid	[76]
*L. rhamnosus*	Ethanol	TCA	-	AEC	[96]
*L. rhamnosus*	Ethanol	Heat treatment + TCA	-	Phenol/sulphuric acid	[97]
*L. rhamnosus +* *S. cerevisiae*	Ethanol	TCA	-	Phenol/sulphuric acid	[98]
*L. paracasei*	Ethanol	-	-	Phenol/sulphuric acid	[75]
*L. plantarum*	Ethanol	-	-	Phenol-sulphuric acid	[115]
*L. plantarum*	Ethanol	TCA	-	Phenol/sulphuric acid	[72]
*L. plantarum*	Ethanol	TCA	-	Phenol/sulphuric acid	[99]
*L. plantarum*	Ethanol	Heat treatment	-	Phenol/sulphuric acid	[71]
*L. kefiranofaciens*	Ethanol	TCA	-	Phenol/sulphuric acid	[100]
*L. kefiranofaciens +* *S. cerevisiae*	Ethanol	-	-	Anthrone reagent	[101]
*L. kefiranofaciens +* *S. cerevisiae*	Ethanol	-	-	Anthrone reagent	[102]
*L. kefiranofaciens +* *S. cerevisiae +*	Ethanol	-	-	Anthrone reagent	[103]
*S. thermophilus*	Acetone	TCA	-	Dry weight	[104]
*S. thermophilus*	Acetone	TCA	-	Gel permeation chromatography	[105]
*S. thermophilus*	Acetone	TCA	-	Dry weight	[106]
*S. thermophilus*	Acetone	TCA	-	Dry weight	[107]
*S. thermophilus*	-	TCA	Ultracentrifugation filtration	HPAEC-PAD	[108]
*S. thermophilus*	Ethanol	TCA	-	Phenol/sulphuric acid	[65]
*S. thermophilus*	Ethanol	-	Ultrafiltration	Phenol/sulphuric acid	[109]
*S. thermophilus*	Ethanol	TCA	Ultrafiltration	Phenol/sulphuric acid	[110]
*S. thermophilus*	Acetone	TCA	-	Dry weight	[111]
*S. thermophilus +* *L. delbrueckii* subsp. *bulgaricus +* *R. rubra*	Acetone + ethanol	TCA	-	Phenol/sulphuric acid	[112]

## Data Availability

Not applicable.
